# Integrin αvβ6 Expression in the Human Pituitary Gland and Pituitary Neuroendocrine Tumors: Immunohistochemical Characterization with Potential Relevance to αvβ6 PET/CT Pituitary Uptake and Theranostic Implications

**DOI:** 10.3390/biom16081182

**Published:** 2026-08-13

**Authors:** Muin Tuffaha, Wael Hananeh, Ehab Shiban, Michael Starke

**Affiliations:** 1 Institute of Pathology, Medical University Lausitz-Carl-Thiem, 03048 Cottbus, Germany; m.tuffaha@mul-ct.de; 2 Department of Veterinary Pathology and Public Health, Faculty of Veterinary Medicine, Jordan University of Science and Technology, Irbid 22110, Jordan; 3 Department of Neurosurgery, Medical University Lausitz-Carl-Thiem, 03048 Cottbus, Germany; e.shiban@mul-ct.de; 4 Department of Nuclear Medicine, Medical University Lausitz-Carl-Thiem, 03048 Cottbus, Germany; m.starke@mul-ct.de

**Keywords:** integrin, pituitary gland, pituitary neuroendocrine, theranostic

## Abstract

Integrins are heterodimeric transmembrane receptors that mediate bidirectional signaling and regulate cell–cell and cell–extracellular matrix interactions. Integrin αvβ6 is an epithelial-associated integrin that has emerged as a promising molecular target for PET/CT imaging using integrin αvβ6-directed radiotracers such as ^68^Ga-Trivehexin, and, most recently, for antibody–drug conjugate therapy in epithelial malignancies. Unexpected physiological and incidental uptake within the pituitary gland has been reported in integrin αvβ6-targeted PET studies, including uptake in morphologically normal pituitary glands and pituitary neuroendocrine tumors (PitNETs). However, the histological basis of integrin αvβ6 expression in the human pituitary gland remains poorly understood. The aim of this study is to characterize the immunohistochemical expression of integrin αvβ6 in normal human pituitary tissue and PitNETs and to evaluate its potential implications for integrin αvβ6-targeted imaging and theranostic applications. Five complete adult pituitary glands obtained at autopsy and 28 PitNETs were examined by immunohistochemistry for integrin αvβ6. Staining distribution, intensity, and cellular localization were assessed in the adenohypophysis, neurohypophysis, and Rathke’s cleft remnants. PitNETs were classified according to transcription factor expression (PIT1, TPIT, and SF1). Among the 28 PitNETs, 17 were SF1-lineage (60.7%), three were PIT1-lineage (10.7), two were TPIT-lineage (7.1%), three lacked a dominant transcription factor (10.7%), and three showed plurilineage expression (10.7%). Integrin αvβ6 expression was evaluated semiquantitatively according to staining intensity and the percentage of positive tumor cells. In normal pituitary glands, integrin αvβ6 immunoreactivity was predominantly membranous and localized to larger adenohypophyseal cells irrespective of transcription factor lineage or hormone phenotype. Strong expression was also observed in the epithelial lining cells of Rathke’s cleft remnants, whereas the neurohypophysis lacked detectable integrin αvβ6 expression. Among the 28 PitNETs, integrin αvβ6 expression was detected in 20 cases (71.4%). Positive tumors demonstrated variable staining intensity and extent, ranging from 20% to 100% positive tumor cells. By lineage, integrin αvβ6 expression was detected in 13 of 17 SF1-lineage tumors (76.5%), one of three PIT1-lineage tumors (33.3%), and zero of two TPIT-lineage tumors (0%). Additionally, all three tumors lacking a dominant transcription factor (100%) and all three plurilineage tumors (100%) demonstrated integrin αvβ6 expression. Eleven integrin αvβ6-positive tumors showed expression in ≥50% of tumor cells, and six exhibited strong or diffuse immunoreactivity. Integrin αvβ6 expression in adenohypophyseal cells and Rathke’s cleft remnants provides a histological explanation for physiological pituitary uptake observed on αvβ6-targeted PET/CT imaging. The high prevalence of integrin αvβ6 expression in PitNETs, particularly in a subset demonstrating strong and diffuse immunoreactivity, suggests potential applicability of integrin αvβ6-targeted molecular imaging and theranostic approaches, including both radioligand- and antibody-based strategies. However, these applications remain investigational and require further validation in preclinical and clinical studies. At the same time, physiological integrin αvβ6 expression in normal anterior pituitary tissue may limit imaging specificity and should be considered when developing integrin αvβ6-targeted radioligand therapies. Further clinicopathological and imaging correlation studies are warranted to define the diagnostic and therapeutic role of integrin αvβ6-targeted approaches in PitNETs.

## 1. Introduction

Integrins are heterodimeric transmembrane receptors composed of α and β subunits. They mediate cell adhesion, migration, extracellular matrix interaction, and intracellular signaling. Integrin αvβ6 is formed by the αv and β6 subunits and is usually described as an epithelial-associated integrin with low expression in most normal adult tissues but increased expression in tissue repair, fibrosis, epithelial malignancies, and tumor progression [1,2]. Its biological relevance is partly related to activation of latent transforming growth factor beta (TGF-β), a pathway involved in fibrosis, epithelial–mesenchymal transition, invasion, and tumor microenvironment remodeling [3,4].

In nuclear medicine, integrin αvβ6 has emerged as a promising target for molecular imaging and theranostics. Several integrin αvβ6-targeting PET tracers, including ^68^Ga-Trivehexin, have entered clinical use, with demonstrated uptake in pancreatic ductal adenocarcinoma, head and neck squamous cell carcinoma, and other epithelial malignancies [5,6,7,8,9,10,11,12]. Most recently, integrin αvβ6-directed targeting has extended beyond diagnostic imaging into antibody–drug conjugate therapeutics: sigvotatug vedotin, a β6-directed antibody–drug conjugate, has shown encouraging efficacy in early-phase trials of non-small cell lung cancer and other solid tumors, underscoring the breadth of clinical interest in this integrin as both a diagnostic and therapeutic target [13,14]. Importantly, early human biodistribution studies of integrin αvβ6 PET tracers reported generally low uptake in most normal tissues, but with notable uptake in the upper gastrointestinal tract and pituitary gland [5,15]. More recently, focal ^68^Ga-Trivehexin uptake has been reported in a morphologically normal pituitary gland, and ^68^Ga-Trivehexin uptake has also been described in pituitary macroadenoma [16,17]. These observations suggest that pituitary uptake on integrin αvβ6-PET/CT may be physiological, pathological, or both.

Despite these imaging observations, the histological basis of integrin αvβ6 uptake in the pituitary gland has not been adequately defined. The pituitary gland contains multiple endocrine cell types in the adenohypophysis, pituicytes and axonal structures in the neurohypophysis, as well as epithelial remnants of Rathke’s pouch. Previous studies of integrins in the pituitary have focused mainly on the integrins α3β1, α6β4, αvβ3, β1, and β3, and extracellular matrix remodeling in pituitary neuroendocrine tumors, rather than integrin αvβ6 specifically [18,19]. Farnoud et al. [18] reported altered integrin expression during adenomatous transformation of the anterior pituitary, including downregulation of α3β1, loss of α6β4, and neoexpression of αvβ3 in a subset of adenomas. This gap is notable because, unlike αvβ3 and the other integrins previously examined in pituitary tissue, αvβ6 is already the target of tracers in active clinical use, making its pituitary expression pattern directly relevant to image interpretation. However, immunohistochemical mapping of integrin αvβ6 in normal pituitary tissue and pituitary adenomas remains largely unexplored.

The current study was initiated from the observation that histological sections of pituitary glands from autopsy cases show marked integrin αvβ6 expression in a subpopulation of large eosinophilic cells in the anterior pituitary and in the epithelial cells of Rathke’s cleft remnants, whereas the neurohypophysis is invariably negative. In addition, 20 out of 28 pituitary neuroendocrine tumors in a preliminary series expressed integrin αvβ6, with varying intensity. These results provide a possible biological rationale for the physiological uptake of integrin αvβ6 PET tracers in the pituitary gland and raise important diagnostic, pathogenetic and therapeutic questions that merit further investigation. Thus, we systematically characterized integrin αvβ6 immunoreactivity in normal pituitary tissue and a well-annotated PitNET cohort, correlating expression with pituitary lineage-defining transcription factors, to establish a histopathological framework for interpreting integrin αvβ6-targeted PET/CT findings in the sellar region.

## 2. Materials and Methods

### 2.1. The Study Design

In this study, integrin αvβ6 immunohistochemical expression was evaluated in normal human pituitary tissue and PitNETs as well as its correlation with pituitary lineage-defining transcription factors, such as pituitary-specific transcription factor 1 (PIT1), T-box pituitary transcription factor (TPIT), and steroidogenic factor 1 (SF1). The goal was to characterize the histopathological and immunophenotypic landscape of integrin αvβ6 expression and to explore its potential relevance as target for integrin αvβ6 PET/CT imaging and integrin αvβ6-targeted radioligand therapy. The study cohort comprised five complete pituitary glands obtained during autopsy from adult individuals and 28 surgically resected PitNETs.

### 2.2. Tissue Samples

Five complete pituitary glands were obtained during autopsy examinations. All specimens were fixed in 10% neutral-buffered formalin and embedded in paraffin. Representative sections included the adenohypophysis, neurohypophysis, and remnants of Rathke’s cleft.

Formalin-fixed, paraffin-embedded tissue blocks from 28 PitNETs resected surgically were retrieved from the pathology archives. Available clinicopathological data included patient age, sex, hormonal activity, radiological tumor size, recurrence status, endocrine laboratory findings, and histopathological diagnosis, including immunohistochemical characterization of pituitary lineage-defining transcription factors (SF1, PIT1, and TPIT).

### 2.3. Immunohistochemistry

Sections 1 µm in thickness were cut from formalin-fixed, paraffin-embedded tissue blocks and mounted on positively charged glass slides. Following deparaffinization and rehydration, heat-induced epitope retrieval was performed using Tris-EDTA buffer (pH 9.0). Endogenous peroxidase activity was blocked using 3% hydrogen peroxide in methanol for 10 min at room temperature prior to incubation with the primary antibodies. Immunohistochemical staining for integrin αvβ6 was performed using a rabbit monoclonal antibody against integrin αvβ6 (clone E4M9P; Cell Signaling Technology, Danvers, MA, USA) at a dilution of 1:200. Antibody binding was visualized using a polymer-based HRP detection system and 3,3′-diaminobenzidine (DAB) chromogen, followed by hematoxylin counterstaining.

Sections from the five pituitary glands were additionally stained with antibodies against the pituitary lineage-defining transcription factors SF1, PIT1, and TPIT. The immunohistochemical staining was performed using the following antibodies: PIT1 (clone EPR28; Abcam; Cambridge, UK; dilution 1:500); TPIT (clone CL6251; Abcam; Cambridge, UK; dilution 1:100) and SF1 (clone H1665; Thermo Fisher; Waltham, MA, USA; dilution 1:100).

All staining was performed in a single experimental run with the same reagents and an automated immunostaining platform in an effort to minimize inter-assay variability. Internal negative controls were neurohypophyseal tissue, vascular endothelial cells and stromal elements as appropriate. The distribution, cellular localization, and staining intensity of integrin αvβ6 immunoreactivity were evaluated and correlated with cellular morphology, the expression of pituitary lineage-defining transcription factors (SF1, PIT1, and TPIT), pituitary cell lineage, and PitNETs subtype according to the fifth edition of the World Health Organization (WHO) Classification of Pituitary Neuroendocrine Tumors (PitNETs) [20]. After IHC slides were prepared, a single pathologist performed a blind evaluation for the entire slides and the results were recorded. During slide evaluation, the scoring pathologist had no access to any clinical data.

### 2.4. Evaluation of Integrin αvβ6 Immunohistochemical Expression

Integrin αvβ6 immunoreactivity was evaluated with respect to staining intensity, extent, and expression pattern. Staining intensity was scored as follows: 0, negative; 1+, weak; 2+, moderate; and 3+, strong. For each case, the predominant staining intensity observed across the entire tumor section at scanning magnification was scored. When multiple intensities were found, the score reflected the intensity observed in the majority of positive tumor cells. The extent of staining was assessed according to the percentage of immunoreactive cells relative to the total tumor cell population in the examined sections, recorded as a continuous variable (e.g., 20%, 50%). The expression pattern was classified as membranous, cytoplasmic, or mixed. Only membranous staining was considered positive, consistent with the known cell surface localization of integrin αvβ6. For PitNETs, cases with ≤5% positive tumor cells were classified as negative.

In normal pituitary glands, integrin αvβ6 expression was assessed separately in adenohypophyseal cell populations using serial sections. Hematoxylin and eosin-stained sections were used to identify cell populations (acidophilic, basophilic, and chromophobic cells) based on their morphology, and correlated with the expression of pituitary transcription factors (PIT1, TPIT, and SF1) on immunohistochemically stained sections. In addition, integrin αvβ6 expression was evaluated in Rathke’s cleft remnants, the neurohypophysis, and vascular and stromal elements. In the PitNET cohort, integrin αvβ6 expression was correlated with tumor lineage, as determined by transcription factor immune-profiling (PIT1, TPIT, and SF1), as well as with tumor cell morphology.

### 2.5. Statistical Analysis

Given the limited sample size, only descriptive statistics were used to summarize the findings. No formal comparative statistical analyses were performed, as the small cohort size particularly for PIT1-lineage and TPIT-lineage tumors precluded meaningful hypothesis testing. Findings regarding integrin αvβ6 expression and clinicopathological parameters in the pituitary neuroendocrine tumor cohort are therefore presented as observed proportions and distributions rather than statistically tested correlations.

## 3. Results

A total of five normal adult pituitary glands obtained at autopsy and 28 PitNETs were evaluated for integrin αvβ6 expression by immunohistochemistry. We analyzed the integrin αvβ6 expression pattern according to pituitary cell lineages, hormone expression and the lineage-specific transcription factors PIT1, TPIT and SF1. Immunohistochemistry was performed on all cases with adequate tissue.

**Figure 1:**
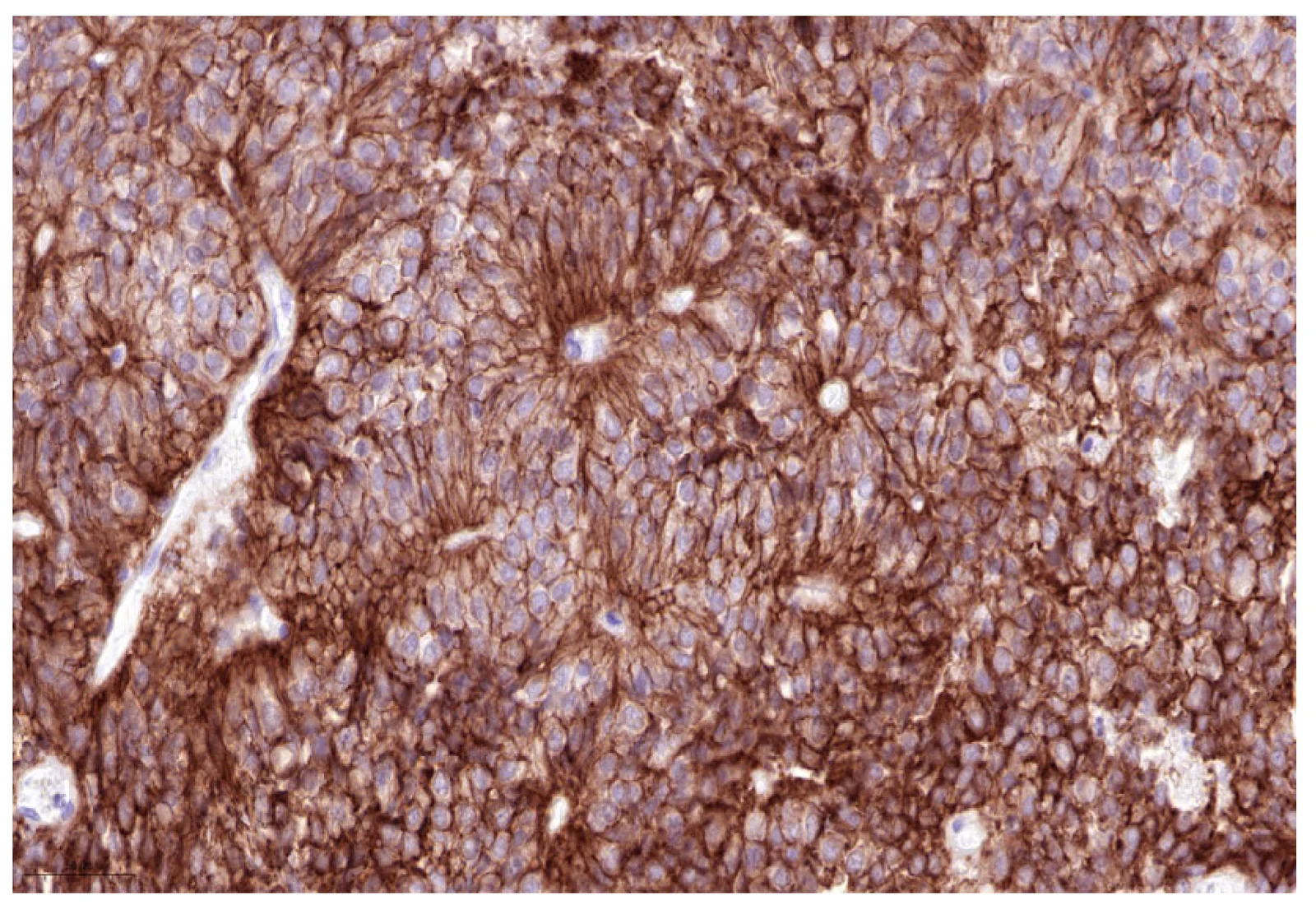
Representative immunohistochemical staining section of pituitary neuroendocrine tumors showing strong membranous integrin αvβ6 expression (immunostain 400×).

Integrin αvβ6 immunoreactivity was predominantly membranous in both normal pituitary tissue and PitNET cells (Figure 1), consistent with the localization of the integrin αvβ6 at the cell surface.

**Figure 2:**
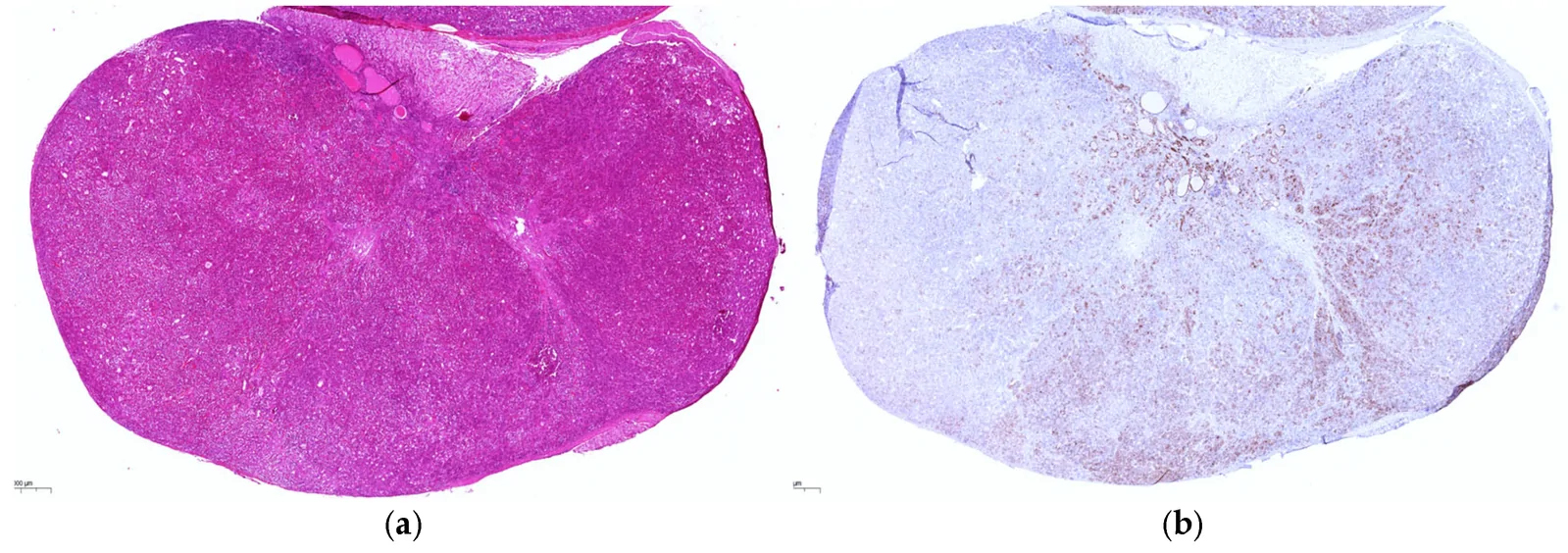

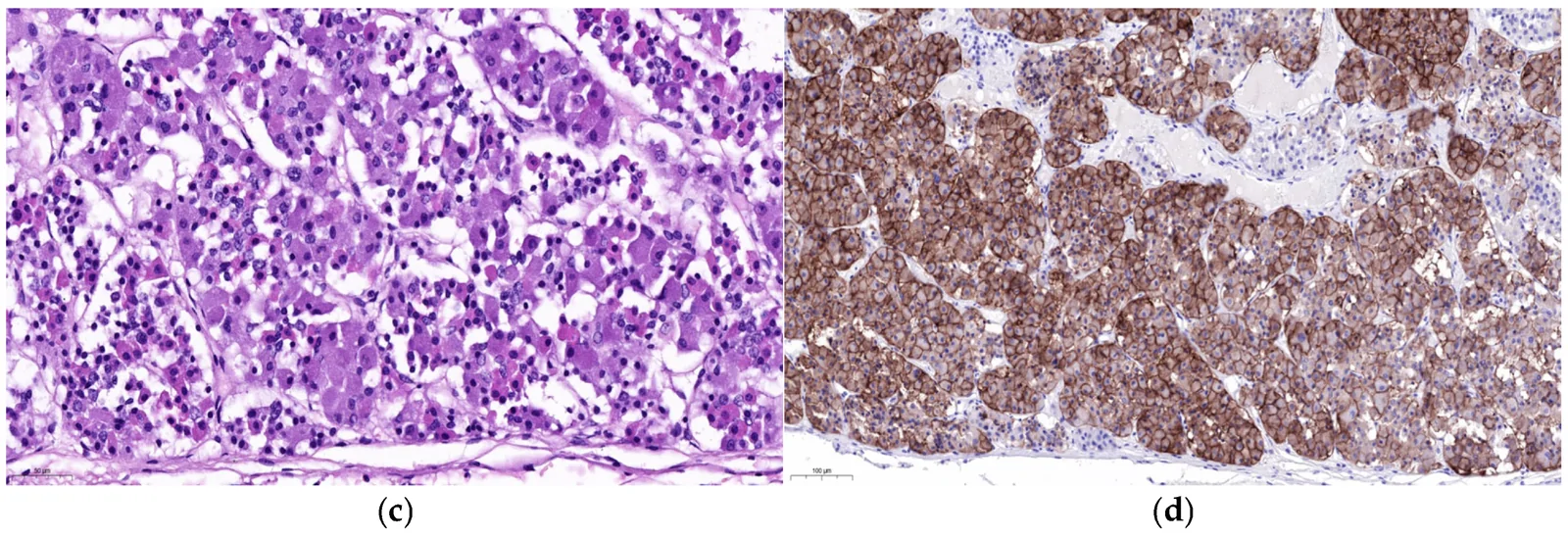
(**a**) Complete section of the pituitary gland, including the adenohypophysis, neurohypophysis, and remnants of Rathke’s cleft located between them (H&E, 20×). (**b**) Immunohistochemical staining of (**a**) for integrin αvβ6 reveals an irregularly distributed population of positive cells, predominantly localized to the anterior and central regions of the gland (immunostain 20×). (**c**) High-power view of (**a**) showing a morphologically heterogeneous cell population (H&E, 100×). (**d**) Immunohistochemical staining of the same section for integrin αvβ6 reveals strong positivity within the population of a subset of adenohypophyseal cells that corresponded to the larger, more cytoplasm-rich cells seen on the matched hematoxylin and eosin-stained sections (immunostain 100×).

In all five normal pituitary glands, integrin αvβ6 expression was observed primarily in a subset of adenohypophyseal cells that corresponded to the larger, more cytoplasm-rich cells seen on the matched hematoxylin and eosin-stained sections. This pattern of staining was independent of pituitary cell lineage and was seen in cells positive for PIT1, TPIT and SF1. In contrast, the majority of the smaller subset of adenohypophyseal cells lacked detectable integrin αvβ6 expression. Thus, integrin αvβ6 expression appeared to be associated with cellular morphology rather than a specific pituitary lineage or hormone phenotype in this descriptive analysis, although the biological significance of this observation remains to be elucidated (Figure 2a–d).

**Figure 3:**
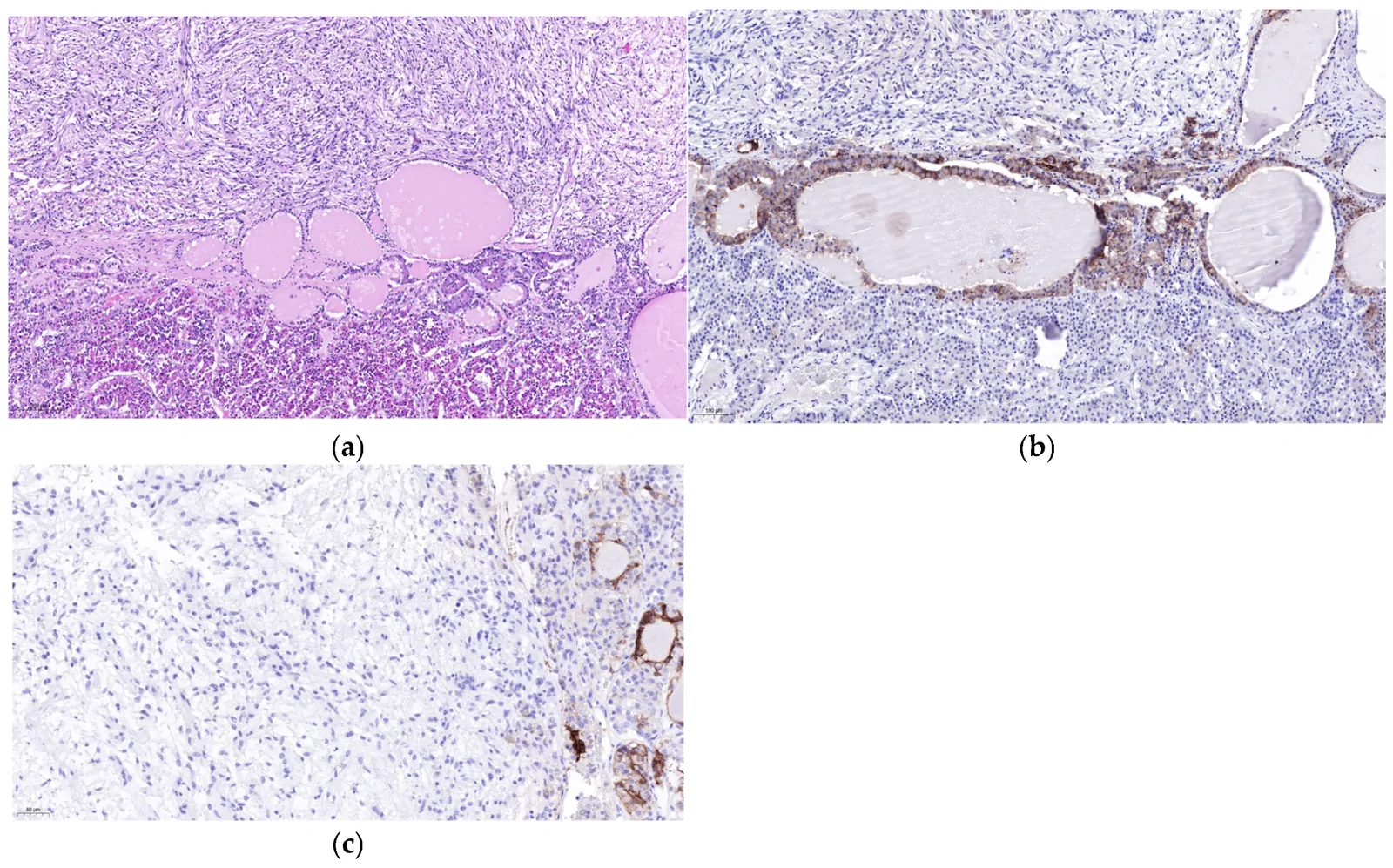
(**a**) Section of the intermediate region of the pituitary gland showing Rathke’s cleft remnants with associated cystic changes (H&E, 40×). (**b**) Immunohistochemical staining of the same section for integrin αvβ6 demonstrates strong immunoreactivity in the epithelial lining of Rathke’s cleft remnants (immunostain 40×). (**c**) High-magnification view of the neurohypophysis showing absence of integrin αvβ6 immunoreactivity (immunostain 100×).

Strong integrin αvβ6 immunoreactivity was also observed in the epithelial lining cells of Rathke’s cleft remnants, whereas no integrin αvβ6 expression was detected in the neurohypophysis (Figure 3a,b).

**Figure 4:**
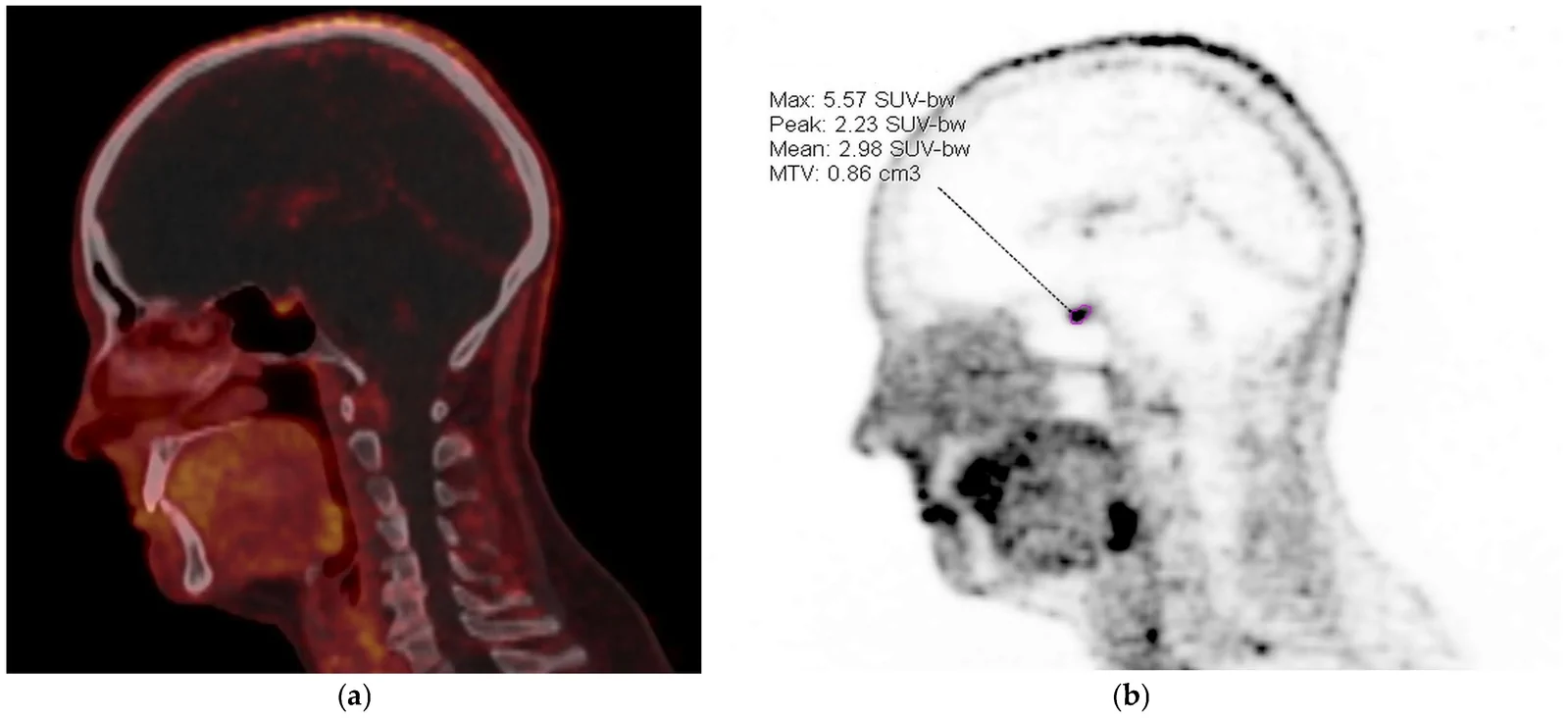
Representative PET/CT images were obtained from a 68-year-old male patient with pancreatic ductal carcinoma. Written informed consent for publication of anonymized images was obtained. (**a**) Sagittal ^68^Ga-Trivehexin(R) PET of neurocranium with marked pituitary tracer uptake. (**b**) Sagittal fused ^68^Ga-Trivehexin (R) PET/CT with marked tracer uptake of the pituitary gland in the sella turcica. The metabolically active lesion is delineated by a volume of interest (VOI) isocontour, with a maximum standardized uptake value normalized to body weight (SUV-bw) of 5.57, peak SUV-bw of 2.23, mean SUV-bw of 2.98, and a metabolic tumor volume (MTV) of 0.86 cm^3^.

The physiological distribution of integrin αvβ6 within the adenohypophysis is consistent with the previously reported physiological uptake of the integrin αvβ6-targeted radiotracer ^68^Ga-Trivehexin in the pituitary gland on PET/CT imaging (Figure 4a,b).

The staining intensity and proportion of integrin αvβ6-positive tumor cells in the PitNET cohort are summarized in Table 1.

Among the 28 PitNETs analyzed, lineage assignment based on pituitary transcription factor expression identified 17 SF1-lineage tumors (60.7%), three PIT1-lineage tumors (10.7%), two TPIT-lineage tumors (7.1%), three tumors lacking a dominant transcription factor expression pattern (10.7%), and three plurilineage tumors showing co-expression of more than one pituitary transcription factor (10.7%).

Overall, integrin αvβ6 immunoreactivity was detected in 20 of 28 PitNETs (71.4%), whereas eight tumors (28.6%) were considered negative according to the predefined cutoff of ≤5% positive tumor cells. Immunoreactivity was predominantly membranous and showed variable staining intensity and extent across the different PitNET subtypes.

**Figure 5:**
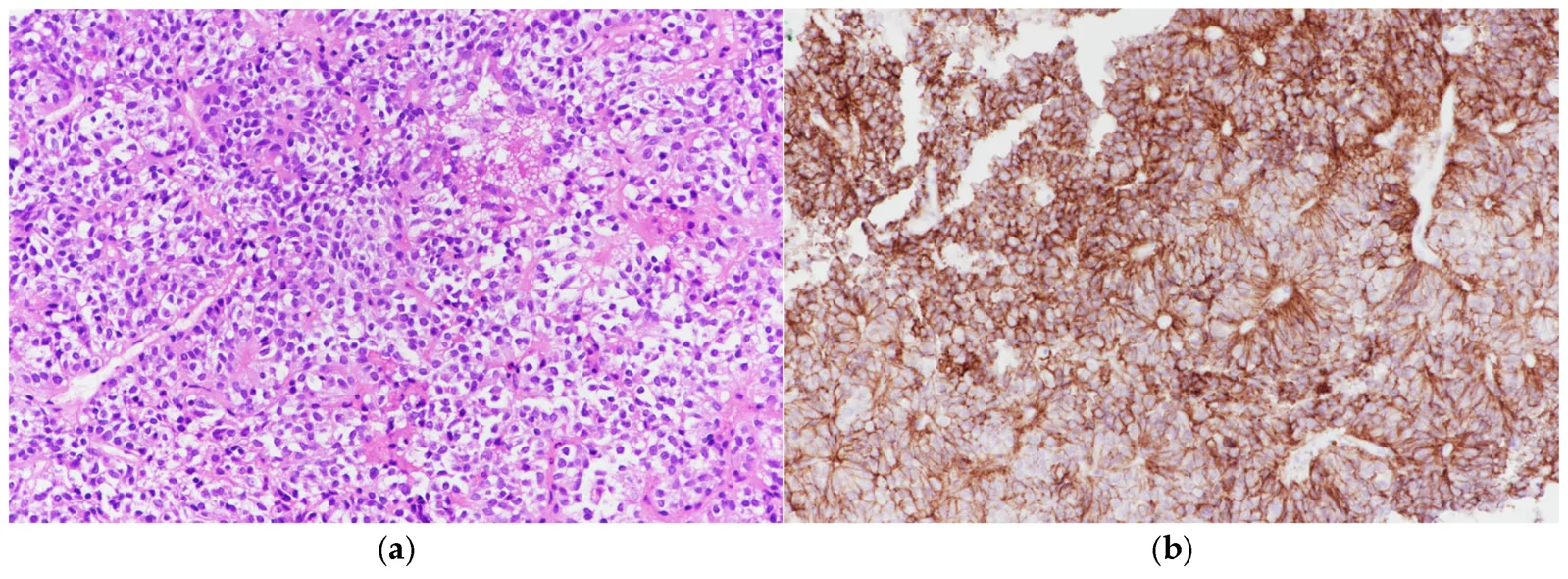
(**a**) Representative histological section of an SF1-lineage pituitary neuroendocrine tumor (H&E, 100×). (**b**) Immunohistochemical staining of the same SF1-lineage demonstrates strong membranous integrin αvβ6 expression in the majority of tumor cells (immunostain 100×).

SF1-lineage tumors constituted the largest subgroup and accounted for the majority of integrin αvβ6-positive cases. Thirteen of 17 SF1-lineage tumors (76.5%) demonstrated integrin αvβ6 expression, with the proportion of positive tumor cells ranging from 20% to 100%. Strong immunoreactivity (+++) was observed in four cases, including tumors with 75–100% positive cells (Figure 5a,b), whereas the remaining positive tumors showed moderate (++) or weak (+) staining. Four SF1-lineage tumors were negative.

Among PIT1-lineage tumors, one of three cases demonstrated integrin αvβ6 expression, showing weak staining involving 50% of tumor cells, whereas the remaining two cases were negative. Both TPIT-lineage tumors lacked detectable integrin αvβ6 expression. Given the small number of PIT1-lineage (*n* = 2) and TPIT-lineage (*n* = 3) tumors in this cohort, any lineage-specific conclusion should be interpreted with caution.

**Figure 6:**
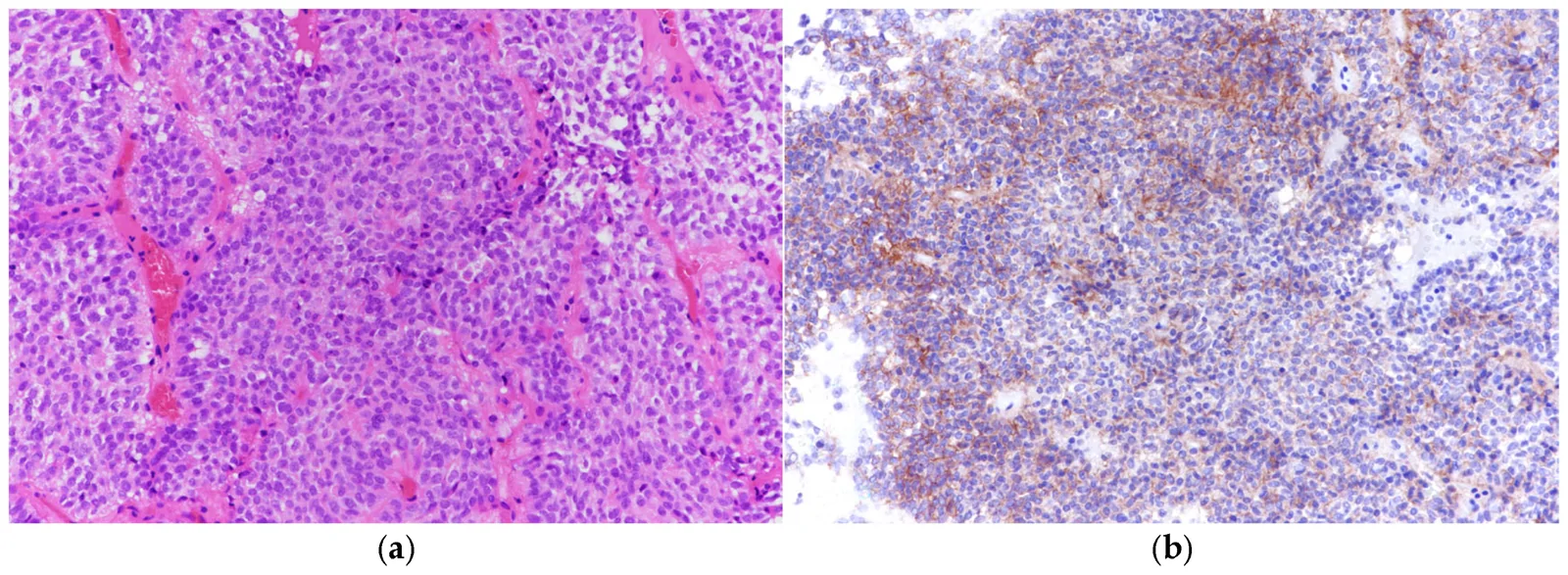
(**a**) Representative histological section of a plurilineage–lineage pituitary neuroendocrine tumor (H&E, 100×). (**b**) Immunohistochemical staining of the same plurilineage reveals weak to moderate integrin αvβ6 expression in approximately 60% of neoplastic cells (immunostain 100×).

Notably, all three tumors lacking a dominant transcription factor expression pattern demonstrated integrin αvβ6 positivity, with moderate staining intensity and 30–50% positive tumor cells. Likewise, all three plurilineage tumors expressed integrin αvβ6, with staining ranging from weak to moderate intensity and involving 20–60% of tumor cells (Figure 6a,b).

**Figure 7:**
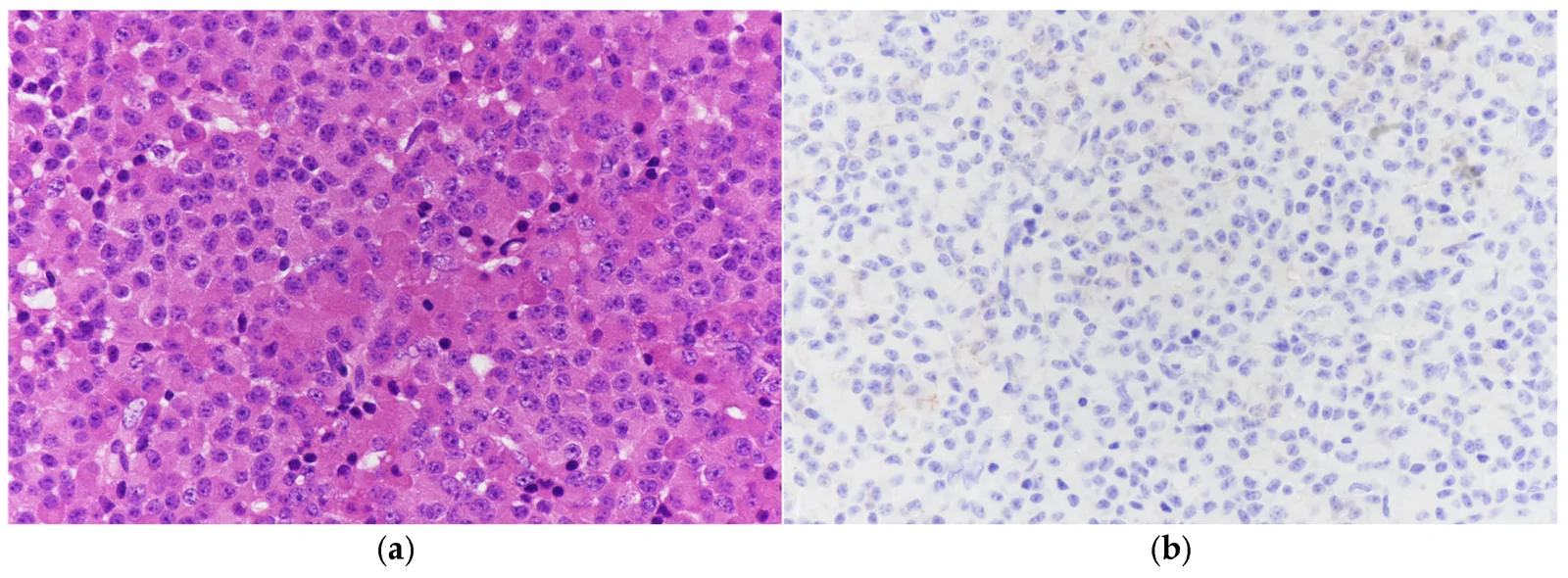
(**a**) Representative histological section of an TPIT-lineage pituitary neuroendocrine tumor (H&E, 200×). (**b**) Immunohistochemical staining of the same TPIT-lineage demonstrates the absence of integrin αvβ6 expression in the majority of tumor cells (immunostain 200×).

The highest levels of αvβ6 expression, defined by both strong staining intensity and a high proportion of positive tumor cells, were observed predominantly among SF1-lineage tumors. However, αvβ6 immunoreactivity was not restricted to a specific transcription factor-defined lineage and was also identified in null-cell and plurilineage tumors. In contrast, all TPIT-lineage tumors (Figure 7a,b) and two of three PIT1-lineage tumors were negative in this cohort.

## 4. Discussion

In the present study, we demonstrate that integrin αvβ6 is physiologically expressed in the human anterior pituitary gland and is frequently expressed in PitNETs. In normal pituitary tissue, integrin αvβ6 immunoreactivity was predominantly membranous and was mainly observed in larger adenohypophyseal cells, without restriction to a specific transcription factor-defined lineage or hormone phenotype. Strong membranous staining was also identified in the epithelial lining of Rathke’s cleft remnants, whereas the neurohypophysis was consistently negative. Among PitNETs, integrin αvβ6 expression was detected in 20 of 28 tumors (71.4%), with variable staining intensity and extent. Expression was most frequently observed in SF1-lineage tumors and was also present in all tumors lacking a dominant transcription factor expression pattern and in all plurilineage tumors, whereas both TPIT-lineage tumors and two of three PIT1-lineage tumors were negative. These findings are broadly compatible with published transcription-factor-based PitNET cohorts, especially the predominance of SF1-lineage tumors. A 2025 single-institution cohort reported SF1-lineage PitNETs as the largest group at 49.5%, followed by PIT1-lineage tumors, TPIT-lineage tumors, null-cell tumors, and plurihormonal/unclassified tumors [21]. This predominance of the SF1/gonadotroph lineage parallels epidemiological data indicating that gonadotroph tumors constitute the majority of clinically non-functioning PitNETs. These lineage-specific observation are descriptive only; the small subgroup sizes particularly for PIT1 and TPIT preclude robust statistical comparisons and require validation in larger, clinically annotated cohorts.

These findings identify integrin αvβ6 as a previously underrecognized biomarker of normal and neoplastic pituitary tissue. Importantly, the membranous staining pattern observed in both adenohypophyseal cells and PitNETs is consistent with the cell surface localization of integrin αvβ6 and is therefore relevant for molecular imaging and ligand-based targeting approaches.

The physiological expression of integrin αvβ6 in the adenohypophysis provides a plausible histopathological explanation for pituitary uptake observed with integrin αvβ6-targeted PET tracers, including ^68^Ga-Trivehexin. It is important to emphasize that this study did not include paired PET/CT and IHC analysis from the same patients. Therefore, the proposed link between integrin αvβ6 IHC expression and in vivo PET tracer uptake demonstrate biological plausibility rather than direct proof and require validation through dedicated correlative studies. Previous imaging study has reported physiological or incidental pituitary uptake in the absence of definite structural abnormalities, as well as uptake in pituitary adenomas [16]. Until now, the biological basis of this finding was unclear. Our immunohistochemical data support the interpretation that at least a significant part of this uptake reflects genuine integrin αvβ6 expression in anterior pituitary tissue rather than nonspecific tracer accumulation.

From a histopathological perspective, the absence of integrin αvβ6 expression in the neurohypophysis is noteworthy and likely reflects the distinct cellular composition of the posterior pituitary, which consists predominantly of axons, pituicytes, and vascular elements rather than epithelial-type endocrine cells. In contrast, the strong expression in Rathke’s cleft remnants is biologically plausible given their epithelial origin from Rathke’s pouch. This finding is diagnostically relevant because Rathke’s cleft cysts or remnants may contribute to integrin αvβ6-related signal in the sellar region and should be considered when interpreting pituitary uptake on integrin αvβ6-targeted imaging.

In PitNETs, integrin αvβ6 expression was not restricted to a single lineage; however, the highest levels of expression were observed predominantly among SF1-lineage tumors. This is of potential clinical interest because SF1-lineage PitNETs largely correspond to gonadotroph tumors, which are commonly clinically non-functioning and frequently present as macroadenomas due to mass effect, accounting for 79% of clinically non-functioning PitNETs [21,22]. The identification of a membranous molecular target in this tumor group may have diagnostic relevance, particularly in selected cases with invasive, recurrent, or clinically silent disease. Nevertheless, given the limited number of PIT1-, TPIT-, null-cell, and plurilineage tumors in the present cohort, lineage-specific conclusions should be interpreted cautiously and require validation in larger series.

Preclinical and early-phase clinical data support the general feasibility of integrin αvβ6-directed radioligand and antibody–drug conjugate strategies in other epithelial tumors strategies using peptide-based ligands labeled with diagnostic radionuclides [23,24,25,26]. Whether such approaches could be extended to PitNETs remains speculative and would require dedicated dosimetric, safety, and efficacy studies given the physiological expression of integrin αvβ6 in normal pituitary tissue.

The theranostic implications of these findings are twofold. First, integrin αvβ6 immunohistochemistry may serve as a tissue-based correlate for integrin αvβ6-targeted PET uptake and could help identify PitNETs with sufficient target expression for molecular imaging. This concept is supported by recent clinical data in non-small cell lung cancer, where tumoral integrin αvβ6 immunohistochemical expression correlated with ^68^Ga-Trivehexin PET uptake and improved nodal staging accuracy relative to ^18^F-FDG PET/CT, providing proof of principle for immunohistochemistry-guided patient selection [27]. Second, the presence of strong and/or diffuse membranous integrin αvβ6 expression in a subset of PitNETs provides a rationale for exploring integrin αvβ6-directed radioligand strategies in carefully selected patients, particularly those with aggressive, recurrent, or treatment-refractory tumors. In the present cohort, several tumors showed expression in at least 50% of tumor cells and a subset demonstrated strong or diffuse immunoreactivity, supporting the concept that integrin αvβ6 may be a relevant molecular target in selected PitNETs or tumors arising from Rathke’s cleft remnants [28,29].

These findings are consistent with and extend a broader histopathology-driven biomarker program characterizing integrin αvβ6 expression across epithelial and neuroendocrine tumor types. Parallel immunohistochemical series from our group have demonstrated predominantly membranous integrin αvβ6 expression amenable to radiotheranostic targeting in triple-negative breast cancer and in gastric and gastroesophageal junction adenocarcinoma [30,31]. Together with the present data, these observations support a unifying model in which integrin αvβ6 immunohistochemistry functions as a practical, tissue-based screening tool that can be applied across diverse tumor types under consideration for integrin αvβ6-directed molecular imaging or therapy.

One major limitation is the physiological expression of integrin αvβ6 in the normal adenohypophysis. Normal anterior pituitary uptake may reduce the specificity of integrin αvβ6 PET/CT for detection of small pituitary lesions and may make it difficult to differentiate physiological uptake, Rathke’s cleft remnants and true neoplastic uptake. Therefore, integrin αvβ6 PET/CT should not replace pituitary MRI, which remains the standard modality for detection, characterization and surgical planning of sellar lesions. Instead, integrin αvβ6 PET/CT may have a complementary role in selected clinical settings, such as interpretation of incidental pituitary uptake during whole-body integrin αvβ6 imaging, assessment of large or recurrent PitNETs or selection of patients for future integrin αvβ6-targeted therapeutic approaches.

The presence of integrin αvβ6 expression in normal adenohypophyseal tissue introduces significant safety concerns for radionuclide therapy targeting integrin αvβ6. The uptake of therapeutic radioligands in normal anterior pituitary tissue could potentially lead to radiation exposure affecting hormone-producing cells, which might result in endocrine issues such as partial or complete hypopituitarism or specific hormone deficiencies. While the lack of integrin αvβ6 expression in the neurohypophysis might imply a reduced risk of posterior pituitary damage, this assumption is speculative and necessitates thorough dosimetric and clinical assessment. Similar effects have been observed with somatostatin receptor-targeted radioligand therapy using ^177^Lu-DOTATATE in neuroendocrine tumors [32]. Our immunohistochemical findings establish a plausible histological basis for pituitary uptake on integrin αvβ6-targeted PET/CT.; however, it is important to emphasize that these data demonstrate association and plausibility rather direct proof of PET signal correlation. Prior to considering integrin αvβ6-targeted radionuclide therapy for PitNETs, future research must establish tumor-to-normal-pituitary-uptake ratios, link immunohistochemical expression with PET signal intensity, and assess the endocrine safety in patients undergoing treatment.

This study has limitations. The cohort size was limited, particularly for PIT1- and TPIT-lineage tumors, and clinical follow-up data were not sufficient to assess associations between integrin αvβ6 expression and tumor aggressiveness, recurrence, or treatment response. Furthermore, although integrin αvβ6 expression was assessed in relation to transcription factor lineage, additional double immunostaining, hormonal phenotypes or spatial analyses would be useful to define more precisely the normal adenohypophyseal cell populations expressing integrin αvβ6.

## 5. Conclusions

This study reveals that integrin αvβ6 is physiologically expressed in the human adenohypophysis and Rathke’s cleft remnants. Integrin αvβ6 is also expressed in the majority of PitNETs (71.4%), with a lineage-associated distribution marked by predominant expression in SF1-lineage tumors and consistent positivity in null-cell and plurilineage neoplasms, whereas both TPIT-lineage tumors and two of three PIT1 lineage tumors were negative. These findings provide a histopathological explanation for the physiological pituitary uptake observed on integrin αvβ6-targeted PET/CT and identify integrin αvβ6 as a potential biomarker for molecular imaging and theranostic applications in PitNETs. However, physiological integrin αvβ6 expression in the normal anterior pituitary must be considered when interpreting integrin αvβ6 PET/CT studies to avoid diagnostic misclassification, and normal pituitary expression may represent a critical dose-limiting factor for integrin αvβ6-targeted radionuclide therapies. Future studies should validate these observations in larger, clinically annotated cohorts, define the specific pituitary cell populations expressing integrin αvβ6, and correlate immunohistochemical expression with in vivo PET/CT uptake to define the diagnostic and therapeutic role of integrin αvβ6-targeted approaches in PitNETs.

## Figures and Tables

**Table 1:** Immunohistochemical expression of integrin αvβ6 in pituitary neuroendocrine tumors (PitNETs) according to transcription factor lineage.

No.	Dominant Pituitary Transcription Factor	Integrin β6 Expression (% Positive Tumor Cells)	Staining Intensity
1	SF1 (90%)	80%	+++
2	SF1 (100%)	90%	++
3	SF1 (50%)	30%	++
4	No dominant transcription factor	30%	++
5	PIT1 (90%)	50%	+
6	SF1 (90%)	75%	+++
7	PIT1 (60%), SF1 (40%)	20%	+
8	PIT1 (80%), SF1 (20%)	60%	++
9	SF1 (80%)	0%	Negative
10	SF1 (100%)	5%	Negative
11	PIT1 (80%)	0%	Negative
12	SF1 (10%)	20%	++
13	SF1 (100%)	50%	++
14	SF1 (50%)	0%	Negative
15	TPIT (100%)	0%	Negative
16	No dominant transcription factor	30%	++
17	TPIT (100%)	0%	Negative
18	PIT1 (100%)	0%	Negative
19	SF1 (25%)	20%	++
20	SF1 (20%)	50%	+
21	SF1 (100%)	50%	++
22	SF1 (100%)	25%	++
23	SF1 (100%)	100%	+++
24	No dominant transcription factor	50%	++
25	PIT1 (75%), TPIT (20%), SF1 (5%)	60%	++
26	SF1 (90%)	0%	Negative
27	SF1 (100%)	90%	+++
28	SF1 (90%)	80%	++

PitNET, pituitary neuroendocrine tumor; PIT1, pituitary-specific transcription factor 1; TPIT, T-box pituitary transcription factor; SF1, steroidogenic factor 1. Staining intensity was scored semiquantitatively as weak (+), moderate (++), or strong (+++). Cases without detectable immunoreactivity were classified as negative. PitNETs with ≤5% integrin expression was considered negative.

## Data Availability

The data presented in this study are available from the corresponding author upon reasonable request.
